# NB compounds are potent and efficacious FOXM1 inhibitors in high-grade serous ovarian cancer cells

**DOI:** 10.1186/s13048-024-01421-4

**Published:** 2024-05-04

**Authors:** Cassie Liu, Makenzie Vorderbruggen, Catalina Muñoz-Trujillo, Sung Hoon Kim, John A. Katzenellenbogen, Benita S. Katzenellenbogen, Adam R. Karpf

**Affiliations:** 1 Eppley Institute, University of Nebraska Medical Center, Omaha, NE 68918-6805 USA; 2 Fred and Pamela Buffett Cancer Center, University of Nebraska Medical Center, Omaha, NE 68918-6805 USA; 3 Department of Chemistry, University of Illinois at Urbana-Champaign, Urbana, IL 61801 USA; 4 Department of Molecular and Integrative Physiology, University of Illinois at Urbana-Champaign, Urbana, IL 61801 USA; 5 Cancer Center, University of Illinois at Urbana-Champaign, Urbana, IL 61801 USA

**Keywords:** Ovarian cancer, High-grade serous ovarian cancer, FOXM1, FOXM1 inhibitors, Fallopian tube epithelial cells, NB compounds, Apoptosis

## Abstract

**Background:**

Genetic studies implicate the oncogenic transcription factor Forkhead Box M1 (FOXM1) as a potential therapeutic target in high-grade serous ovarian cancer (HGSOC). We evaluated the activity of different FOXM1 inhibitors in HGSOC cell models.

**Results:**

We treated HGSOC and fallopian tube epithelial (FTE) cells with a panel of previously reported FOXM1 inhibitors. Based on drug potency, efficacy, and selectivity, determined through cell viability assays, we focused on two compounds, NB-73 and NB-115 (NB compounds), for further investigation. NB compounds potently and selectively inhibited FOXM1 with lesser effects on other FOX family members. NB compounds decreased FOXM1 expression via targeting the FOXM1 protein by promoting its proteasome-mediated degradation, and effectively suppressed FOXM1 gene targets at both the protein and mRNA level. At the cellular level, NB compounds promoted apoptotic cell death. Importantly, while inhibition of apoptosis using a pan-caspase inhibitor rescued HGSOC cells from NB compound-induced cell death, it did not rescue FOXM1 protein degradation, supporting that FOXM1 protein loss from NB compound treatment is specific and not a general consequence of cytotoxicity. Drug washout studies indicated that FOXM1 reduction was retained for at least 72 h post-treatment, suggesting that NB compounds exhibit long-lasting effects in HGSOC cells. NB compounds effectively suppressed both two-dimensional and three-dimensional HGSOC cell colony formation at sub-micromolar concentrations. Finally, NB compounds exhibited synergistic activity with carboplatin in HGSOC cells.

**Conclusions:**

NB compounds are potent, selective, and efficacious inhibitors of FOXM1 in HGSOC cells and are worthy of further investigation as HGSOC therapeutics.

**Supplementary Information:**

The online version contains supplementary material available at 10.1186/s13048-024-01421-4.

## Background

High-grade serous ovarian cancer (HGSOC) is the most common and deadliest subtype of epithelial ovarian cancer [1]. Two limitations of HGSOC clinical management are its late diagnosis, after disease spread, and the absence of a curative therapy for recurrent disease. Patients with HGSOC need more therapy options, which requires identification of novel therapeutic targets and the development of new therapies. Forkhead Box M1 (FOXM1) is a master transcriptional regulator of oncogenic phenotypes in many cancers, including HGSOC, and has been identified as a potential cancer therapeutic target [2–7]. Notably, the FOXM1 pathway is aberrantly activated in 87% of HGSOC cases, making it the most activated oncogenic pathway in this disease [8, 9]. Additionally, the expression and activation of FOXM1 is linked to poor prognosis in ovarian cancer [10, 11]. FOXM1 is widely overexpressed and hyperactivated in HGSOC and other cancers, which is driven by mechanisms including copy number gain, disrupted p53 and Rb signaling, and activation by oncogenic kinases [7, 9, 12]. Mounting evidence supports a multifunctional role for FOXM1 in HGSOC, including the promotion of cell proliferation, cell invasion, metastasis, chemotherapy resistance, cancer stemness, genomic instability, and altered metabolism [7].

Over the last decade, several small molecule inhibitors (SMI) of FOXM1 have been identified and characterized [13]. Among these agents, thiostrepton and Forkhead Domain Inhibitor 6 (FDI-6) have been the most extensively studied. Thiostrepton is a thiazole antibiotic and complex natural product that promotes FOXM1 degradation with high potency [14–16]. Notably, thiostrepton also inhibits the ribosome and proteosome, indicating a pleiotropic mechanism of action [16–18]. In contrast to thiostrepton, FDI-6 is a small molecule that disrupts the DNA binding function of FOXM1 to its canonical binding motif [19]. Because FOX family members share a conserved DNA binding domain (DBD), the degree of selectivity of FDI-6 for FOXM1 remains uncertain. Moreover, FOXM1 has additional functions aside from binding to canonical FOX motifs that might limit the anticancer action of FDI-6, including binding to non-canonical sites, participating in transcriptional complexes without directly binding DNA, and binding of other oncoproteins to regulate their activity [20–25]. Other recently reported FOXM1 inhibitors include monensin [26], N-phenylphenanthren-9-amine [27], and RCM-1 [28]. The mechanisms of action of these agents against FOXM1, and their anti-cancer activities have not been studied extensively.

Recently, we reported a novel class of FOXM1 inhibitors possessing a 1,1-diarylethylene core structure (named NB compounds). These compounds include NB-55 (monoamine), NB-73 (diamine methiodide salt), and NB-115 (diamine methiodide salt). These compounds directly bind to purified FOXM1 protein as well as to FOXM1 in cells [29]. Furthermore, NB compounds promoted FOXM1 protein degradation in triple negative breast cancer (TNBC) cells, induced apoptosis in TNBC cells, inhibited TNBC proliferation with low micromolar potency, and reduced TNBC xenograft growth in immunodeficient mice [29]. HGSOC has a highly similar molecular and genomic profile as TNBC, including ubiquitous *TP53* mutations, frequent Rb pathway dysregulation, frequent impairment in the homologous recombination (HR) DNA repair pathway, and extensive somatic copy number alterations [30]. In addition, we previously reported that TNBC and HGSOC have virtually identical frequencies of *FOXM1* copy number gains and FOXM1 overexpression [12]. Based on the genetic similarities between TNBC and HGSOC, along with the activity of NB compounds in TNBC, we hypothesized that NB compounds may target FOXM1 and possess anticancer activity in HGSOC cells.

Here we report that, compared with other FOXM1 inhibitors (including thiostrepton and FDI-6), NB-73 and NB-115 have a superior combination of potency, efficacy, and selectivity for HGSOC cells as compared to control fallopian tube epithelial (FTE) cells, the progenitor cells of HGSOC [31, 32]. NB compounds potently inhibited FOXM1 in HGSOC cells, suppressed FOXM1 target genes, induced apoptotic cell death, and synergized with carboplatin, the standard of care chemotherapy for HGSOC [1]. These data encourage investigation of NB compound FOXM1 inhibitors as therapeutic agents for HGSOC.

## Methods

### Cell culture

CAOV3 and OVCAR5 cell lines were a gift from Dr. Anirban Mitra (Indiana University). CAOV3, OVCAR5, OVCAR8 (National Cancer Institute Division of Cancer Treatment and Diagnosis Cell Line Repository, Bethesda, MD, USA), and COV318 (Sigma-Aldrich, St. Louis, MO, USA) cell lines were cultured in DMEM (Corning, Corning, NY, USA) supplemented with 10% fetal bovine serum (FBS) (Gibco, Fisher Scientific, Waltham, MA, USA) and 1% penicillin–streptomycin (pen-strep) (Gibco). OVCAR4 (National Cancer Institute Division of Cancer Treatment and Diagnosis Cell Line Repository) were cultured in RPMI-1640 (Gibco) supplemented with 10% FBS and 1% pen-strep. The human immortalized FTE cell line FT282, a gift from Dr. Ronny Drapkin (University of Pennsylvania) [33], was used to generate the clonal cell line FT282-C11 as previously described [34], and was cultured in DMEM and Ham’s F12, 50/50 mix (Corning) supplemented with 10% FBS and 1% pen-strep. All cell lines were maintained at 37 °C in a humidified incubator with 5% CO_2_. Cell lines were authenticated using short tandem repeat (STR) analysis at the DNA Services Facility, University of Illinois at Chicago. Cell lines were confirmed mycoplasma-free by PCR analysis using Mycofind™ Mycoplasma PCR Detection Kit (Clongen Laboratories, LLC, Gaithersburg, MD, USA).

### Chemical compounds

Details on the preparation and spectroscopic characterization of NB compounds were previously described [29]. NB-73, NB-115, Thiostrepton (Sigma-Aldrich #598226), FDI-6 (Sigma-Aldrich #SML1392), RCM-1 (R&D Systems, Minneapolis, MN, USA, #6845), *N*-phenylphenanthren-9-amine (Sigma-Aldrich, St. Louis, MO, USA, #761966), MG132 (Sigma-Aldrich #474790), Q-VD-OPh (R&D Systems #OPH001), and olaparib (Selleck Chemicals, Houston, TX, USA, #S1060) were dissolved in DMSO. NB-55 and monensin (R&D Systems #5223) were dissolved in ethanol. Carboplatin (Sigma-Aldrich #C2538) was dissolved in water. All compounds were stored at -20 °C.

### Protein extractions

Whole cell proteins were extracted using radio-immunoprecipitation assay (RIPA) buffer (1X PBS, 1% NP-40, 0.5% sodium deoxycholate, 0.1% sodium dodecyl sulfate [SDS]) supplemented with protease and phosphatase inhibitors (Sigma-Aldrich). Briefly, media was removed from cells, cells were washed in ice cold PBS, and cells were lysed for 15 min in RIPA buffer, collected from the plate, and sonicated. The resulting extracts were centrifuged at 4 °C for 10 min at 16,100 × *g* to remove cell debris. In some cases (e.g., when measuring apoptotic markers such as cleaved PARP) floating cells were isolated in parallel and combined with the remainder of the cell sample prior to protein extraction. Protein concentrations were determined using the Pierce™ BCA Protein Assay Kit (Fisher Scientific, Waltham, MA, USA).

Nuclear and cytoplasmic protein fractions were extracted using the NE-PER Nuclear and Cytoplasmic Extraction Reagents (ThermoFisher Scientific, Waltham, MA, USA). Protein was isolated following the manufacturer’s protocol including supplementation of the CER I and NER reagents with protease and phosphatase inhibitors (Sigma-Aldrich). Protein concentrations were determined as described above.

### Western blotting

Equivalent amounts of protein per well, as determined by BCA assays, were loaded into Invitrogen™ NuPAGE™ 4–12%, Bis–Tris, 1.5 mm, mini protein gels (Fisher Scientific) or 7.5% Mini-Protein TGX precast protein gels (Bio-Rad, Hercules, CA, USA), and subsequently transferred to 0.45 µm polyvinylidene difluoride (PVDF) membranes (Millipore Sigma, St. Louis, MO, USA), using a wet transfer system. Membranes were stained with ThermoFisher Scientific™ Pierce™ Reversible Protein Stain Kit (Fisher Scientific) or Ponceau S (Acros Organics, Fisher Scientific, Waltham, MA, USA) to confirm equivalent protein loading. Membranes were blocked with 5% nonfat dry milk (Kroger, Cincinnati, OH, USA) in TBS-T at room temperature. Membranes were incubated in primary antibodies in 5% BSA (Sigma-Aldrich). Primary antibodies included anti- FOXM1 (Cell Signaling Technology, CST, Danvers, MA, USA) (CST #5436, 1:1,000), p-FOXM1 (Thr600) (CST #14655, 1:1,000–1:2,000), AURKB (Abcam, Waltham, MA, USA, #2254, 1:1,000), CCNB1 (CST #4138, 1:1,000–1:2,000), CDC25B (CST #9525, 1:1,000–1:2000), PLK1 (CST #4513, 1:1,000) FOXA1 (CST #53528, 1:1,000–1:2,000)), FOXK2 (CST #12008, 1:1,000), FOXO3a (CST #12829, 1:10,000), cleaved PARP (cl-PARP) (CST #5625, 1:1,000), ubiquitin (CST #14049, 1:20,000). alpha-tubulin (CST #2144, 1:5,000), β-actin (Santa Cruz Biotechnology, Dallas, TX, USA, # 47778, 1:10,000), and Lamin B1 (CST #12586, 1:1,000). Following primary antibody incubation, membranes were washed in TBS-T at room temperature and then incubated in goat anti-rabbit secondary antibody (CST #7074, 1:500–1:5,000) or horse anti-mouse secondary antibody (CST #7076, 1:500–1:10,000) in 5% non-fat dry milk (Kroger) in TBS-T for 1 h at room temperature. SuperSignal™ West Pico PLUS Chemiluminescent Substrate (Fisher Scientific) was used for protein detection. Ultra blue X-ray films (Light Labs, Aurora, CO, USA) were applied to the blots, and subsequently developed in a standard film processor. Quantification of protein expression was performed using Fiji software [35].

### CyQuant assay

Cells were seeded into 96-well plates and treated with serial dilutions with the designated compounds for 72 h. Medium was then removed from the wells, and the cells were frozen at -80 °C for storage. After thawing, cell viability was analyzed using the CyQUANT™ Cell Proliferation Assay for cells in culture (Invitrogen, ThermoFisher Scientific, Waltham, MA, USA), according to manufacturer’s instructions. The CyQuant assay utilizes a DNA binding fluorescent dye, and the signal is proportional to the number of live cells, which thus reflects the effect of drug treatment on both cell proliferation and cytotoxicity. To integrate these two measures (proliferation and cytotoxicity) into one simple term, we refer to CyQuant data as a measure of cell viability. Fluorescence intensity was measured using POLARstar OPTIMA microplate multi-detection plate reader (BMG LabTech, Cary, NC, USA) with settings specified by the manufacturer’s instructions.

### RT-qPCR

RNA was extracted using TRIzol Reagent (Invitrogen) and purified with the Direct-zol™ RNA MiniPrep Kit (Zymo Research, Irvine, CA, USA) following the manufacturer’s protocol with DNase treatment. RNA integrity was confirmed by running denatured samples on agarose gels in MOPS buffer containing formaldehyde. RNA yield and purity was assessed using a Nanodrop 2000 instrument (ThermoFisher) and by determining 260/280 and 260/230 nm sample absorbance. cDNA was generated using 200–1,000 ng RNA with the High-Capacity cDNA Reverse Transcription kit (Applied Biosystems, ThermoFisher Scientific, Waltham, MA, USA) following the manufacturer’s protocol. cDNA was diluted 1:5 in PCR-grade water (Sigma) and 1.0 μl sample was added to a mix of iTaq Universal SYBR® Green Supermix and primers. PCR primer sequences are listed in Table 1. Reaction mixtures were run using the CFX Connect Real-Time System (Bio-Rad) with an annealing temperature of 60^O^C for 40 cycles. Standard curves were generated using products from endpoint RT-PCR purified by gel-purification (QIAquick PCR purification kit, Qiagen, Germantown, MD, USA). mRNA measurement for genes of interest were normalized to 18S rRNA.

**Table 1:** RT-qPCR primers

Human gene/mRNA	Orientation	Oligonucleotide Sequence (5’ to 3’)
*18 s rRNA*	Forward	CAGCCACCCGAGATTGAGCA​
*18 s rRNA*	Reverse	TAGTAGCGACGGGCGGTGTG​
*FOXM1*	Forward	GCAGGCTGCACTATCAACAA​
*FOXM1*	Reverse	TCGAAGGCTCCTCAACCTTA​
*CCNB1*	Forward	AACTTTCGCCTGAGCCTATTTT​
*CCNB1*	Reverse	TTGGTCTGACTGCTTGCTCTT​
*SKP2*	Forward	GGTGTTTGTAAGAGGTGGTATCGC​
*SKP2*	Reverse	CACGAAAAGGGCTGAAATGTTC​
*CDC25B*	Forward	CCTCCGAATCTTCTGATGCAG​
*CDC25B​*	Reverse	GCGTCTGATGGCAAACTGC​

### Time course analysis of FOXM1 mRNA and protein expression after NB compound treatment

CAOV3 cells were seeded into 6-well or 35 mm plates and treated with DMSO or NB-73. RNA and protein were isolated in parallel from untreated samples at the time of treatment (0 h) and at time points after treatment. RNA was isolated and used for RT-qPCR analysis. Whole cell protein extracts were isolated and used for western blotting.

### Drug washout study

To assess FOXM1 protein expression, cells were seeded into 6-well plates and treated with DMSO or NB compounds the next day. At 3- or 6-h post-treatment, media was aspirated and cells were washed with PBS and incubated with drug-free media until reaching 24, 48, or 72 h post the initial treatment. At time points post-treatment, protein was isolated from washout and no washout (i.e., continuous treatment) treatment conditions and used for western blotting. To assess the effects of drug washout on cell viability, cells were seeded in 96-well plates, treated as described above, and cell viability was assessed at 72 h post the initial treatment using CyQuant assays.

### Incucyte analysis

CAOV3 cells were seeded into 96-well plates in DMEM media containing Incucyte Nuclight Rapid Red Dye (Sartorius, Bohemia, NY, USA, Cat #4717) 1:1,000 (for live cell nuclear labeling) and Incucyte Cytotox Green Dye (Sartorius Cat #4633) 250 μM (for counting of dead cells). The following day, cells were treated with DMSO or NB compounds at 0.1, 0.5 or 1.0 μM concentrations. After treatment, cells were incubated in a Incucyte S3 time lapse imager (Sartorius). We obtained 5 pictures per well every 2 h, for a total of 72 h. Data were analyzed and plotted using Incucyte Live-cell Imaging and Analysis software.

### Cell cycle analyses

Cells were seeded into 6-well plates and treated with the designated compound(s) for 24 h. For cell collection, the adherent cells were trypsinized, centrifuged at 500 × g to form a pellet, and washed with PBS. For cell fixation, the washed cells were centrifuged at 500 × *g* to form a pellet, PBS was aspirated from the cell pellet, and 1.0 mL 70% ice-cold ethanol was slowly added dropwise while the cell pellet was gently vortexed. Cells were stored in -20 °C for at least 3 h. For cell staining, the fixed cells were washed with 4.5 mL PBS thrice, centrifuging at 2,000 × *g* to form a pellet each time, and the final cell pellet was resuspended in 200 µL FxCycle™ PI/RNase Staining Solution (ThermoFisher Scientific). The cells were incubated for 30 min, protected from light. Cells were transferred to a 1.5 mL microcentrifuge tube for analysis. Cell cycle was analyzed by flow cytometry using the Cell Cycle program on the Guava® Muse® Cell Analyzer. A stained sample was used to adjust instrument settings, determine gating strategies, and define the DNA profile content histogram. Experimental samples were thoroughly resuspended before loading onto the Guava® Muse® Cell Analyzer, and 10,000 events were acquired for each experimental sample.

### Caspase-3/7 activity assay

Cells were seeded into 6-well plates and treated with the designated compounds until the desired time point. Floating cells and trypsinized cells from the wells were collected, centrifuged at 500 × *g*, and washed with PBS. Cells were prepared using the Muse® Caspase-3/7 Kit (Luminex Corporation, Austin, TX, USA) according to the manufacturer’s instructions. Cells were analyzed by flow cytometry using the Guava® Muse® Cell Analyzer (Luminex Corporation).

### Two-dimension (2D) and three-dimension (3D) colony formation assay

For 2D anchorage-dependent colony formation assays, 2500 cells in single-cell suspension were seeded into triplicate wells of 6-well plates, incubated for 24 h, then treated with the designated compounds and allowed to form colonies for 7 days (OVCAR4, OVCAR5, OVCAR8) or 14 days (CAOV3). At the end of incubation period, cells were fixed in methanol, stained with crystal violet, washed with water, and air-dried overnight. Pictures of the wells were captured and colonies were counted using Count and Plot Histograms of Colony Size (countPHICS) software [36], a macro written for ImageJ. The threshold for colony size was automatically determined by countPHICS.

For 3D anchorage-independent colony formation assays, 2500 cells in single-cell suspension were seeded into a 0.4% SeaPlaque™ Agarose (Lonza Bioscience, Rockland, ME, USA) liquid solution and placed on top of a solidified layer of 0.8% SeaPlaque™ Agarose solution in triplicate wells of 6-well plates. The top layer with cells was allowed to solidify at room temperature for at least 30 min. After 24 h, cells were treated with the designated compounds in the media above the two agarose layers and allowed to form colonies for 14 days. Following incubation, cells were stained with crystal violet and washed with water. Pictures of the wells were taken, and colonies were counted using countPHICS software [36]. The threshold for colony size was automatically determined by countPHICS.

### Drug synergy assessment

CyQuant assays were performed as described above. Concentrations of each compound were decided based on the diagonal method to measure drug synergy and are presented in Table 2 [37]. Drug interactions and synergy assessment was determined using CompuSyn software (ComboSyn, Inc., Paramus, NJ, USA).

**Table 2:** Drug Concentrations used for synergy testing

NB-73 or NB-115	Carboplatin	Olaparib
16.4 nM	0.36 µM	1.6 µM
41 nM	0.9 µM	4.1 µM
102 nM	2.3 µM	10.2 µM
256 nM	5.7 µM	25.6 µM
640 nM	14 µM	64 µM
1.6 µM	35 µM	160 µM
4 µM	88 µM	400 µM
10 µM	222 µM	1000 µM

For simultaneous drug treatment, cells were seeded into 96 well plates at 2500 cells per well and 24 h later the media was removed and treated with NB compounds and carboplatin. 72 h later, media was removed, and the plates were transferred to -80° C. Later, plates were thawed and processed for CyQuant assays. For sequential drug treatment, cells were seeded into 96 well plates at 2500 cells per well, and 24 h later the media was removed and treated with NB compounds. 24 h later, the media was removed, and cells were treated with carboplatin. 48 h later, the media was removed, plates were transferred to -80° C. Later, plates were thawed and processed for CyQuant assays.

### Study replication and statistics

Experiments were performed using three biological replicates, typically each with technical triplicate measurements. In some cases, representative data from one replicate are presented (e.g., western blot images). Statistical analyses were performed using GraphPad Prism and specific tests used are provided in Figure Legends. If the numerical *p*-values are not specifically indicated, the significance notation used is * for *p* < 0.05, ** for *p* < 0.01, *** for *p* < 0.001, and **** for *p* < 0.0001.

## Results

### Potency, efficacy, and tumor cell selectivity of FOXM1 inhibitors in HGSOC cells

We investigated the effect of eight previously reported FOXM1 inhibitors on HGSOC cell viability. We utilized CAOV3 and OVCAR4 cells, which are validated to model HGSOC both in vitro and in vivo [38, 39], and measured the effect on cell viability using CyQuant assays. The data are summarized in Table 3 (drugs ordered alphabetically). Of the eight tested compounds, two had low potency (FDI-6, NP9) and one had no observable activity (RCM-1) on HGSOC cells. Monensin had high potency but low efficacy in one cell line (OVCAR4). Thiostrepton showed high potency and efficacy. The three diarylethene compounds (NB compounds) also showed high potency and efficacy, with NB-73 and NB-115 outperforming NB-55.

**Table 3:** Potency and efficacy of FOXM1 inhibitors in HGSOC cell lines^a^

Compound	HGSOC cell line			
	CAOV3		OVCAR4	
	Potency [IC_50_ (µM)]	% Efficacy (maximum dose)	Potency [IC_50_ (µM)]	% Efficacy (maximum dose)
FDI-6^b^	9.1	61% (20 μM)	4.7	61% (20 μM)
Monensin	0.10	100% (10 μM)	0.024	52% (10 μM)
NB-55	3.0	89% (10 μM)	3.2	96% (10 μM)
NB-73	0.60	98% (10 μM)	0.34	86% (10 μM)
NB-115	0.49	100% (10 μM)	0.56	85% (10 μM)
N-phenylphenanthren- 9-amine (NP9)	23	85% (100 μM)	39	100% (100 μM)
RCM-1^c^	n/d^d^	0% (50 μM)	n/d^d^	0% (50 μM)
Thiostrepton	0.62	93% (2.5 μM)	0.98	99% (2.5 μM)

^a^The experimental endpoint was cell viability, using CyQuant assay. Potency was defined as the IC_50_ value expressed in μM. Efficacy was defined as the % loss of cell viability at the maximum tested drug concentration

^b^The fluorescence of FDI-6 partially interferes with the CyQuant assay, which has minor effects on the values reported

^c^RCM-1 treated cells showed no loss of viability up to the highest tested concentration of 50 μM

^d^Not Determined

**Fig. 1:**
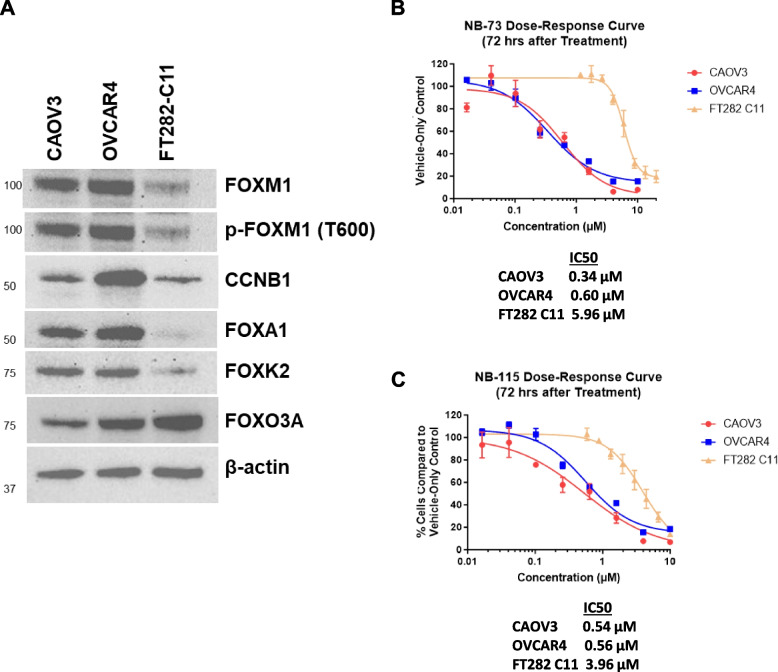
Protein expression and NB compound dose response in HGSOC and FTE cells. **A** Western blot analyses of FOXM1, FOXM1-P (P-Threonine 600), CCNB1, FOXA1, FOXK2, and FOXO3a expression in CAOV3 (HGSOC), OVCAR4 (HGSOC), and FT282-C11 (immortalized FTE cells). β-actin staining is a shown as a protein loading control. **B-C** Dose–response curves of CAOV3 (red), OVCAR4 (blue), and FT282-C11 (yellow) cells treated with **B** NB-73 or **C** NB-115 for 72 h. Cell viability was analyzed by CyQuant assay. Three independent trials were performed with three technical replicates per trial. Values represent mean ± SEM. Curves were created using nonlinear regression with least squares (ordinary) fit in GraphPad Prism. IC50 values are indicated below the graphs

To investigate the cancer cell selectivity of FOXM1 inhibitors, we utilized the immortalized (FTE) cell line FT282-C11 as non-malignant, cell of origin matched control [33, 34]. FT282-C11 cells have low expression of FOXM1, FOXM1-P (activated FOXM1), CCNB1 (canonical FOXM1 target), FOXA1, and FOXK2 (all oncoproteins), and elevated FOXO3a (a tumor suppressor), as compared to HGSOC cells (Fig. 1A), validating their utility as a normal control. We tested a sub-set of FOXM1 inhibitors, including the three NB compounds and the two most widely used FOXM1 inhibitors, thiostrepton and FDI-6. The data summarized in Table 4 (drugs ordered alphabetically) revealed that NB-73 and NB-115 have higher selectivity (approximately tenfold) for HGSOC cells compared to the other three compounds, which exhibit little selectivity. Representative CyQuant data for NB-73 and NB-115 are shown in Fig. 1B and C. We further validated the activity of NB-73 and NB-115 on two additional HGSOC cell lines, OVCAR5 and OVCAR8 (Fig S1). Thus, based on our analysis of FOXM1 inhibitor potency, efficacy, and selectivity, we chose NB-73 and NB-115 for further study.

**Table 4:** Selectivity of FOXM1 inhibitors in HGSOC cells vs. FTE cells^a^

	Cell Line						
Compound	CAOV3		OVCAR4		FT282-C11		Fold HGSOC cell selectivity^c^
	IC_50_ (µM)	Efficacy	IC_50_ (µM)	Efficacy	IC_50_ (µM)	Efficacy	
FDI-6^b^	9.1	61%	4.7	61%	11	76%	1.6
NB-55	3.0	89%	3.2	96%	2.8	96%	0.90
NB-73	0.60	98%	0.34	86%	6.0	81%	13
NB-115	0.49	100%	0.56	85%	3.8	100%	7.2
Thiostrepton	0.62	93%	0.98	99%	0.73	100%	0.91

^a^Potency and efficacy were determined using the CyQuant assay, as described in Table 3

^b^The fluorescence of FDI-6 partially interferes with the CyQuant assay, which has minor effects on the values reported

^c^The fold HGSOC cell selectivity was calculated by dividing the IC_50_ value in FT282-C11 by the averaged IC_50_ values of CAOV3 and OVCAR4 cells

### NB compounds suppress FOXM1, FOXM1-P, and FOXM1 targets in HGSOC cell lines.

**Fig. 2:**
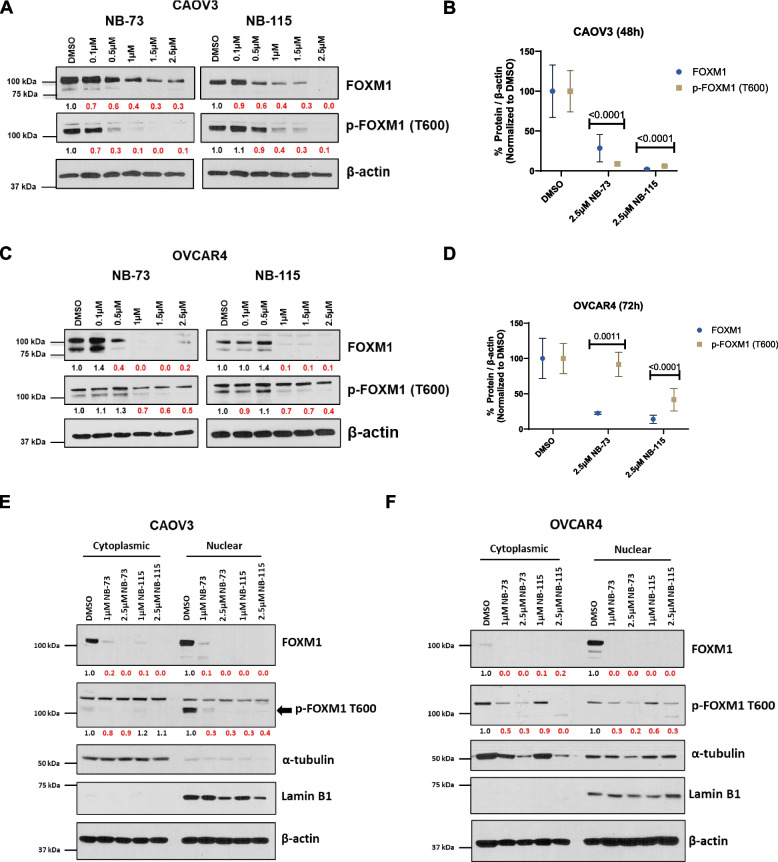
NB compound treatment suppresses FOXM1 and FOXM1-P expression in HGSOC cells. **A** Western blot analysis of FOXM1, FOXM1-P, and β-actin (loading control) expression in CAOV3 cells treated with the indicated concentrations of NB-73 or NB-115 for 48 h. Numerical values below the images indicate protein expression normalized by β-actin, and red numbers indicate reduced expression relative to DMSO control. **B** Quantified western blot data from panel **A**, with data from three biological replicates plotted. Two-way ANOVA with Dunnett’s multiple comparisons test, error bars indicate mean ± SD. **C-D** As in panels **A**-**B**, but in OVCAR4 cells treated for 72 h. **E** Western blot analysis of FOXM1, FOXM1-P, Lamin B1 (nuclear isolation control), α-tubulin (cytoplasm isolation control), and β-actin (overall loading control) expression in cytoplasmic and nuclear protein fractions from CAOV3 cells treated with the indicated concentrations of NB-73 and NB-115 for 48 h. Numerical values below the images indicate protein expression normalized to β-actin and red numbers indicate reduced expression relative to the DMSO control. **F** as in **E**, except in OVCAR4 cells treated for 72 h

We measured FOXM1 protein expression as the primary molecular pharmacodynamic (PD) endpoint, as NB compounds are known to directly bind to FOXM1, promoting its degradation [29]. In addition, we measured a transcriptionally active form of FOXM1, FOXM1-pThr600 (i.e., FOXM1-P), to infer the effect of drug treatment on the FOXM1 pathway [40, 41]. In both cell lines, NB compounds, at low micromolar concentrations, suppressed FOXM1 and FOXM1-P expression at 24-, 48, and 72-h post-treatment. We observed the most robust FOXM1 suppression at the 48-h time point in CAOV3 cells and the 72-h time point in OVCAR4 cells (Fig. 2A-D).

Next, we examined whether NB compounds suppress nuclear FOXM1 expression, which is likely the biologically active fraction of FOXM1, by quantifying protein expression in the nuclear and cytosolic compartments of HGSOC cells. Notably, in both HGSOC cell lines, NB compounds suppressed FOXM1 in both the cytosolic and nuclear compartments (Fig. 2E-F). FOXM1-P was enriched in the nuclear compartment, particularly in CAOV3 cells, and was suppressed by NB compound treatment (Fig. 2E-F).

**Fig. 3:**
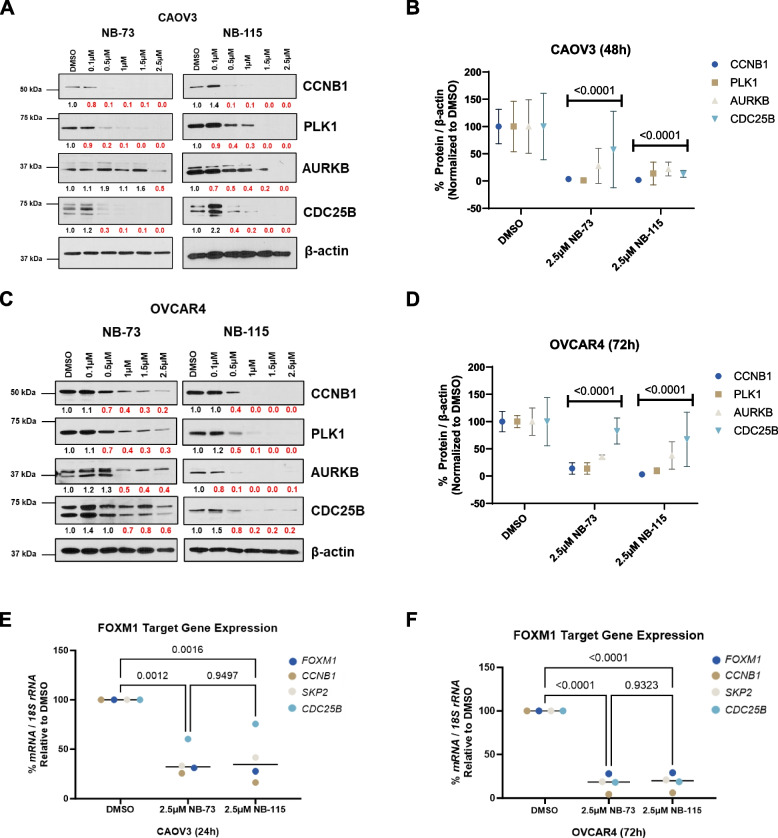
NB compound treatment suppresses FOXM1 target protein and gene expression in HGSOC cells. **A** Western blot analysis of FOXM1 targets and β-actin expression in CAOV3 cells treated with the indicated concentrations of NB-73 or NB-115 for 48 h. Numerical values below the images indicate protein expression normalized by β-actin and red numbers indicate reduced expression relative to DMSO control. **B** Quantified western blot data from panel **A**, data from three biological replicates are plotted. Two-way ANOVA with Dunnett’s multiple comparisons test, error bars indicate mean ± SD. **C-D** As in panels **A**-**B**, but in OVCAR4 cells treated for 72 h. **E** RT-qPCR analysis of FOXM1 target gene expression in CAOV3 cells treated with the indicated concentration of NB-73 or NB-115 for 24 h. Line indicates median, two-way ANOVA with Tukey’s multiple comparison test. **F** As in panel **E**, except in OVCAR4 cells treated for 72 h

Next, we measured the effect of NB compound treatment on the protein expression of FOXM1 target genes. We analyzed four canonical FOXM1 targets: Cyclin B1 (CCNB1), PLK1, AURKB, and CDC25B [42–44]. As seen for FOXM1 and FOXM1-P, low micromolar treatments with NB-73 or NB-115 suppressed FOXM1 targets at the protein level in both cell lines (Fig. 3A-D). Because FOXM1 promotes the gene expression of its targets, we also utilized RT-qPCR to measure the expression of four well characterized FOXM1 mRNA targets following NB compound treatment (*FOXM1*, *CCNB1*, *SKP2*, *CDC25B*). *FOXM1* was included in these genes as FOXM1 is reported to regulate its own gene expression [15]. The data showed that NB compound treatment causes suppressed of FOXM1 target genes in both cell lines (Fig. 3E-F).

**Fig. 4:**
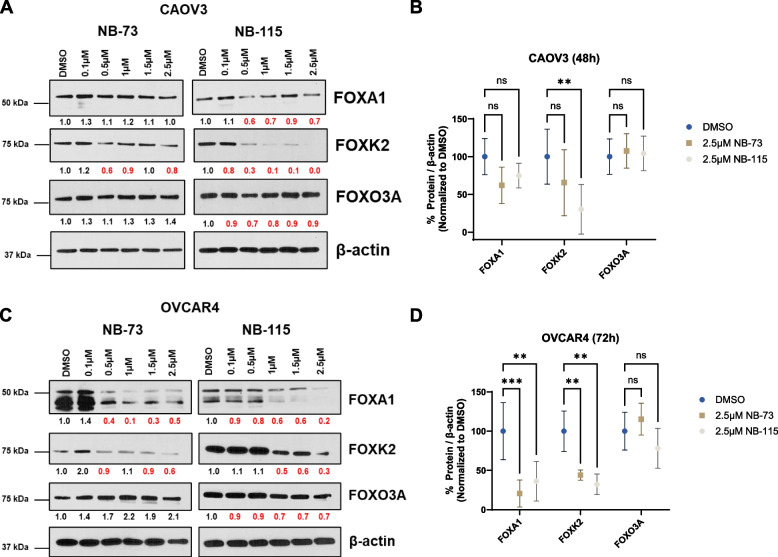
NB compound treatment effect on expression of FOX family members in HGSOC cells. **A** Western blot analysis of the indicated FOX proteins and β-actin expression in CAOV3 cells treated with the indicated concentrations of NB-73 or NB-115 for 48 h. Numerical values below the images indicate protein expression normalized by β-actin and red numbers indicate reduced expression relative to the DMSO control. **B** Quantified western blot data from panel **A**, data from three biological replicates are plotted. Two-way ANOVA with Dunnett’s multiple comparisons test, error bars indicate mean ± SD. **C-D** As in panels **A**-**B**, but in OVCAR4 cells treated for 72 h

FOXM1 is a member of the forkhead (FOX) transcription factor family, which has ~ 50 members unified by a conserved DBD but consists of proteins of diverse function [45]. Thus, an important characteristic of any FOXM1 inhibitor is its selectivity within the FOX protein family. To test this in the context of NB compound treatment of HGSOC cells, we used western blotting to determine the expression of three additional FOX family members: FOXA1, FOXK2, and FOXO3a. FOXA1 and FOXK2 function as oncoproteins in ovarian cancer [46–48], while, in contrast, FOXO3a is a tumor suppressor [49]. Remarkably, NB compound treatment suppressed FOXA1 (significantly in OVCAR4, trend apparent in CAOV3) and FOXK2, with no significant effect on FOXO3a (Fig. 4). It should be noted that NB compound suppression of FOXA1 and FOXK2 was less consistent and occurred at higher drug concentrations as compared to their effect on FOXM1 (Figs. 2 and 4). Nevertheless, the data indicate, for the first time, that NB compounds can suppress other oncogenic FOX family members.

### NB compounds target FOXM1 protein and promote FOXM1 proteasomal degradation in HGSOC cell lines

**Fig. 5:**
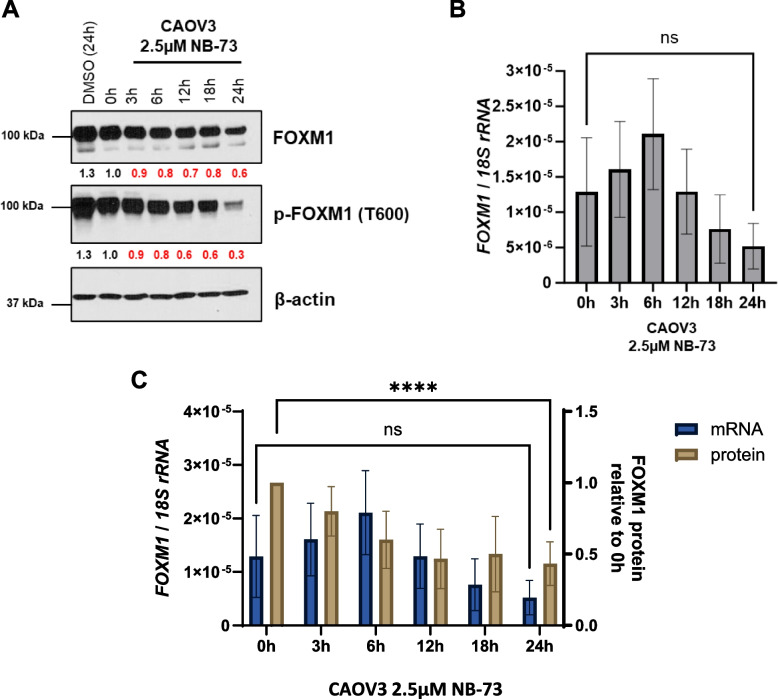
NB-73 treatment suppresses FOXM1 protein prior to *FOXM1* mRNA in CAOV3 cells. **A** Western blot analysis of FOXM1, FOXM1-P, and β-actin expression in CAOV3 cells treated with 2.5 μM NB-73 for the indicated time points. **B** RT-qPCR of *FOXM1* gene expression in CAOV3 cells treated with 2.5 μM NB-73 for the indicated time points. Error bars indicate mean ± SD, unpaired t-test. **C** FOXM1 protein and *FOXM1* mRNA from three independent experiments as described in panels **A** and **B**, plotted together. Error bars indicate mean ± SD, two-way ANOVA with Dunnett’s multiple comparisons test

**Fig. 6:**
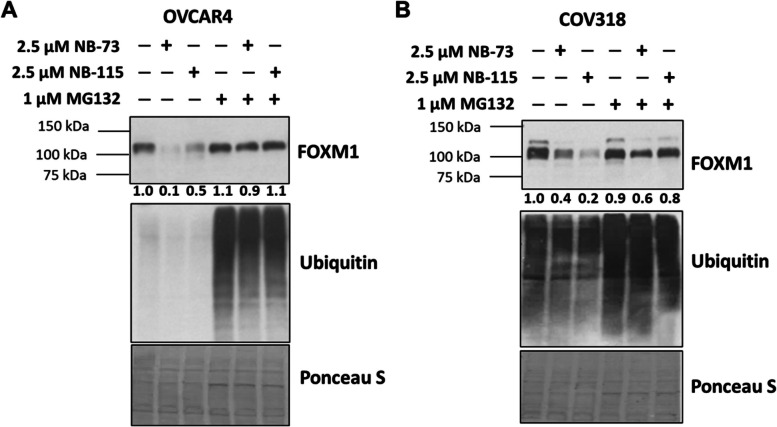
NB compound treatment mediated FOXM1 suppression in HGSOC cells is rescued by MG132 co-treatment.** A** Western blot analysis of FOXM1 and total ubiquitin expression in OVCAR4 cells treated with the indicated compounds for 24 h. Protein quantification is provided below each protein band, and protein expression is normalized to DMSO control (lane 1). Ponceau S staining shows total protein loading. Protein levels were quantified using Fiji software [35]. **B** As in panel **A**, except in COV318 cells treated for 24 h

The observation that NB compounds suppressed FOXM1 at both the protein and mRNA levels (Figs. 2 and 3E-F) led us to further investigate the mechanism of action (MOA) of NB compounds in HGSOC cells. We hypothesized that, following treatment with NB compounds, FOXM1 protein suppression would precede *FOXM1* mRNA suppression. To test this, we conducted a kinetic study in which we measured FOXM1 mRNA and protein at time points up to 24 h in CAOV3 cells. As hypothesized, FOXM1 protein was suppressed prior to *FOXM1* mRNA and was apparent as early as 3 h post-treatment (Fig. 5). These data are in agreement with studies showing that NB compounds target FOXM1 protein in breast cancer cells [29]. Consistently, co-treatment of NB compounds with the proteasome inhibitor MG132 rescued FOXM1 protein suppression in HGSOC cell lines (Fig. 6).

### NB compound treatment leads to sustained suppression of FOXM1 in HGSOC cell lines

**Fig. 7:**
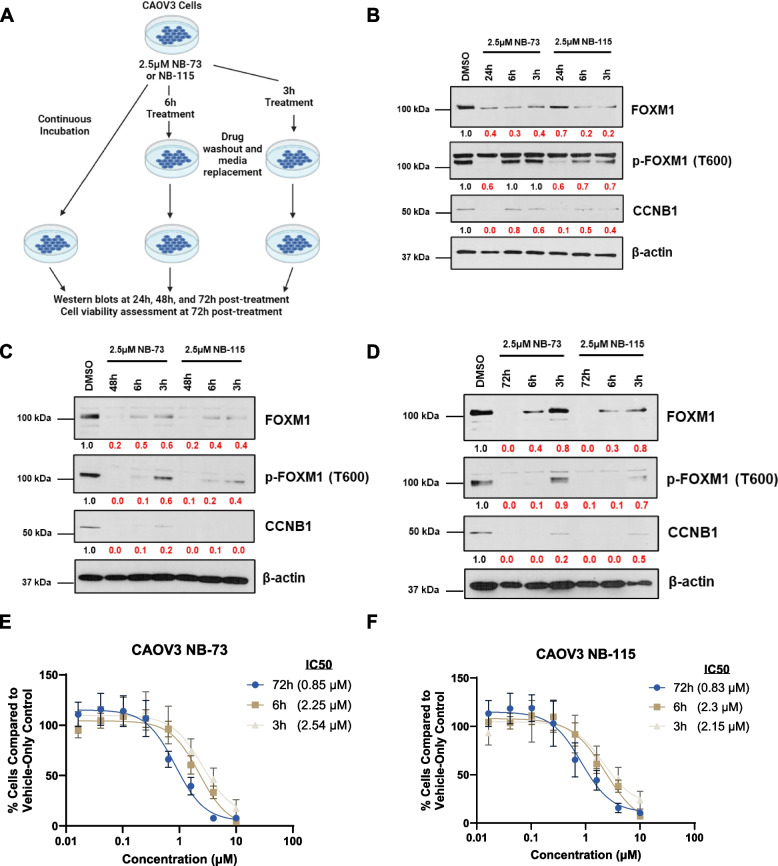
NB compound treatment mediated FOXM1 suppression in CAOV3 cells after drug washout. **A** Experimental schematic, created with BioRender. **B-D** Western blot analysis of FOXM1, FOXM1-P, CCNB1, and β-actin expression in CAOV3 cells treated as indicated in panel **A**, for **B** 24 h, **C** 48 h, and **D** 72 h post-treatment. **E** CyQuant cell viability assay data for CAOV3 cells treated with NB-73. The three curves compare data from no washout (72 h), 6 h drug exposure then washout (6 h), and 3 h drug exposure then washout (3 h). IC50 values for each condition are indicated in brackets. Error bars indicate mean ± SD, non-linear regression curve. **F** As in **E**, except for NB-115 treatment

An important PD parameter is the length of time of a drug’s effect after drug removal. We sought to determine whether NB compound treatment has a sustained effect on FOXM1 in HGSOC cells by conducting drug washout studies [50]. We treated CAOV3 cells with NB compounds using three different regimens: 1) no drug washout (i.e., treatment followed by continuous drug incubation), 2) drug washout after 6 h of incubation, or 3) drug washout after 3 h of incubation (Fig. 7A). Notably, the suppressive effects of NB compounds on FOXM1 were largely maintained for 72 h after drug washout (Fig. 7B-D). To further assess the durability of NB compound effects on HGSOC cells, we determined NB compound IC50 values in CAOV3 cells treated with the three different treatment regimens (Fig. 7A). We observed a 2–threefold decrease of NB compound potency with drug washout, supporting that the effects of NB compounds are largely sustained in HGSOC cells for up to 72 h after drug removal (Fig. 7E-F). Additionally, we confirmed the sustained effect of NB compounds on FOXM1 suppression and cell viability using OVCAR4 cells (Fig S2).

### Kinetic analysis of cell proliferation and cell death following NB compound treatment

**Fig. 8:**
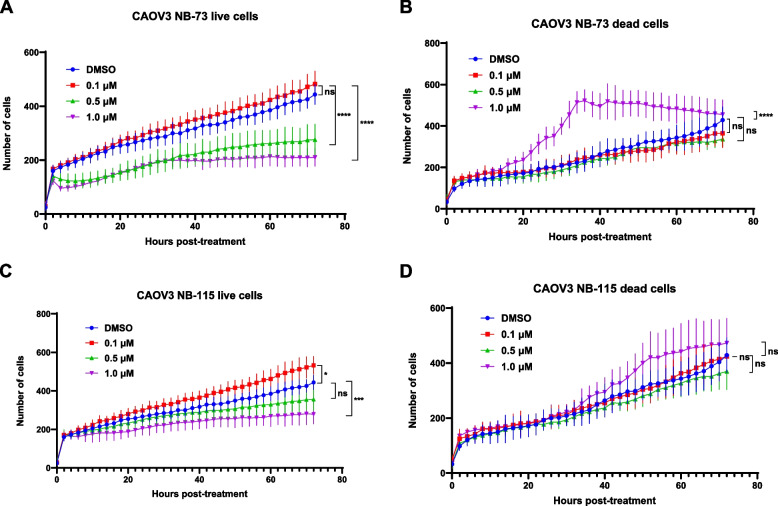
Kinetic analysis of live and dead CAOV3 cells after NB compound treatment. **A** Live and **B** dead CAOV3 cells following treatment with DMSO (vehicle) or the indicated concentrations of NB-73 over 72 h, quantified using the Incucyte platform. **C-D** As in panels **A**-**B**, except for CAOV3 cells treated with NB-115. Live cell count is a measure of cell proliferation, while dead cell count is a measure of cell death. *P*-values were calculated using one-way ANOVA with Sidak correction for multiple comparisons

To investigate the cell physiological effects of NB compounds in HGSOC cells over time, we used the Incucyte platform to perform simultaneous quantitative kinetic analysis of live cells (i.e., cell proliferation) and dead cells (i.e., cell death) following NB compound treatment of CAOV3 cells. We selected three drug concentrations for study (0.1, 0.5, and 1.0 μM) based on the drug potency observed in CyQuant cell viability assays (Table 3). CAOV3 cells treated with NB-73 showed a dose-dependent suppression of cell proliferation, which began within 2–4 h post-treatment in cells treated with 0.5 μM or 1.0 μM NB-73 (Fig. 8A). Notably, cells treated with 1.0 μM NB-73, but not the lower drug concentrations, exhibited a marked increase in cell death by ~ 20 h post-treatment (Fig. 8B). Similar results were obtained for NB-115 treatment (Fig. 8C-D). Surprisingly, in this assay, 0.1 μM NB-115 showed increased proliferation compared to the DMSO control (Fig. 8C). This might reflect hormesis, an adaptive response of cells to moderate stress, which is widely described in pharmacology [51]. In summary, at doses below their IC50 value, NB compounds appear to mainly inhibit HGSOC cell proliferation, while at doses above their IC50 value, the major effect is cell death. This finding agrees with observations in breast cancer cells, in which cell death was the principal outcome of NB compound treatment [29].

### NB compounds alter HGSOC cell cycle in a cell line dependent manner

We next investigated the effect of NB compound treatment on HGSOC cell cycle profiles using flow cytometry analysis of propidium iodide-stained cells. We performed the analyses 24 h after treatment with NB-73 or NB-115, using both CAOV3 and OVCAR4 cells. As shown in Fig S3A, in CAOV3 cells treatment with NB compounds did not lead to significant changes in cell cycle distribution, although a trend was apparent towards reduction of cells in S phases with small increases in G1 and G2 cells. In contrast, OVCAR4 showed significant increases in G1 populations along with some decreases in S phase cells (Fig S3B). However, the modest nature of the observed changes in both cell lines suggests that altered cell cycle is not the major cellular outcome of NB compound treatment in HGSOC cells.

### NB compound treatment promotes apoptotic cell death in HGSOC cells

**Fig. 9:**
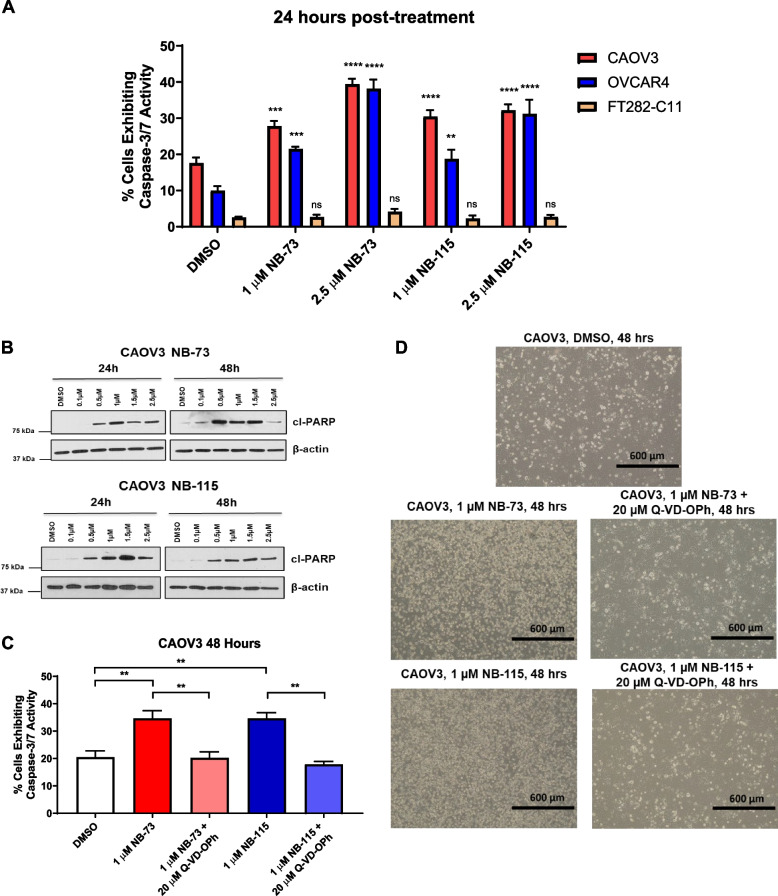
NB compound treatment promotes apoptosis in HGSOC cells. **A** Caspase 3/7 activity in CAOV3, OVCAR4, and FT282-C11 cells treated with DMSO or the indicated concentrations of NB compounds for 24 h. Assays were run in three independent trials. Values represent mean ± SEM and *p*-values denote comparison to DMSO control. **B** Western blot analysis of cl-PARP and β-actin expression in CAOV3 cells treated with NB-73 or NB-115, for indicated drug concentrations and time points. **C** Caspase 3/7 activity in CAOV3 cells treated with DMSO or the indicated compounds for 48 h. **D** Representative images of CAOV3 cells treated with DMSO, NB-73 or NB-115 alone, or NB compounds concurrently with the pan-caspase inhibitor Q-VD-OPh for 48 h. Cells are shown at 10X magnification, and the black bar represents 600 µm

To investigate the mechanism by which NB compounds cause HGSOC cell death, we measured the activity of caspases 3 and 7, which are activated during apoptosis [52]. In both CAOV3 and OVCAR4 cells treated with NB-73 and NB-115, but not in FT282-C11 cells, there was a robust increase in caspase 3/7 activity, supporting an apoptotic mechanism (Fig. 9A). In agreement, NB compounds caused the induction of cleaved PARP (cl-PARP), another biomarker of apoptosis [53] (Fig. 9B). Most notably, co-treatment of CAOV3 cells with NB compounds and the pan-caspase inhibitor Q-VD-OPh [54] inhibited caspase 3/7 activity (as expected) and rescued the cells from drug-induced cytotoxicity (Fig. 9C and D). Together, these data implicate apoptosis as a major effect of NB compounds on HGSOC cells.

### FOXM1 suppression is independent of apoptosis in NB compound treated HGSOC cells

**Fig. 10:**
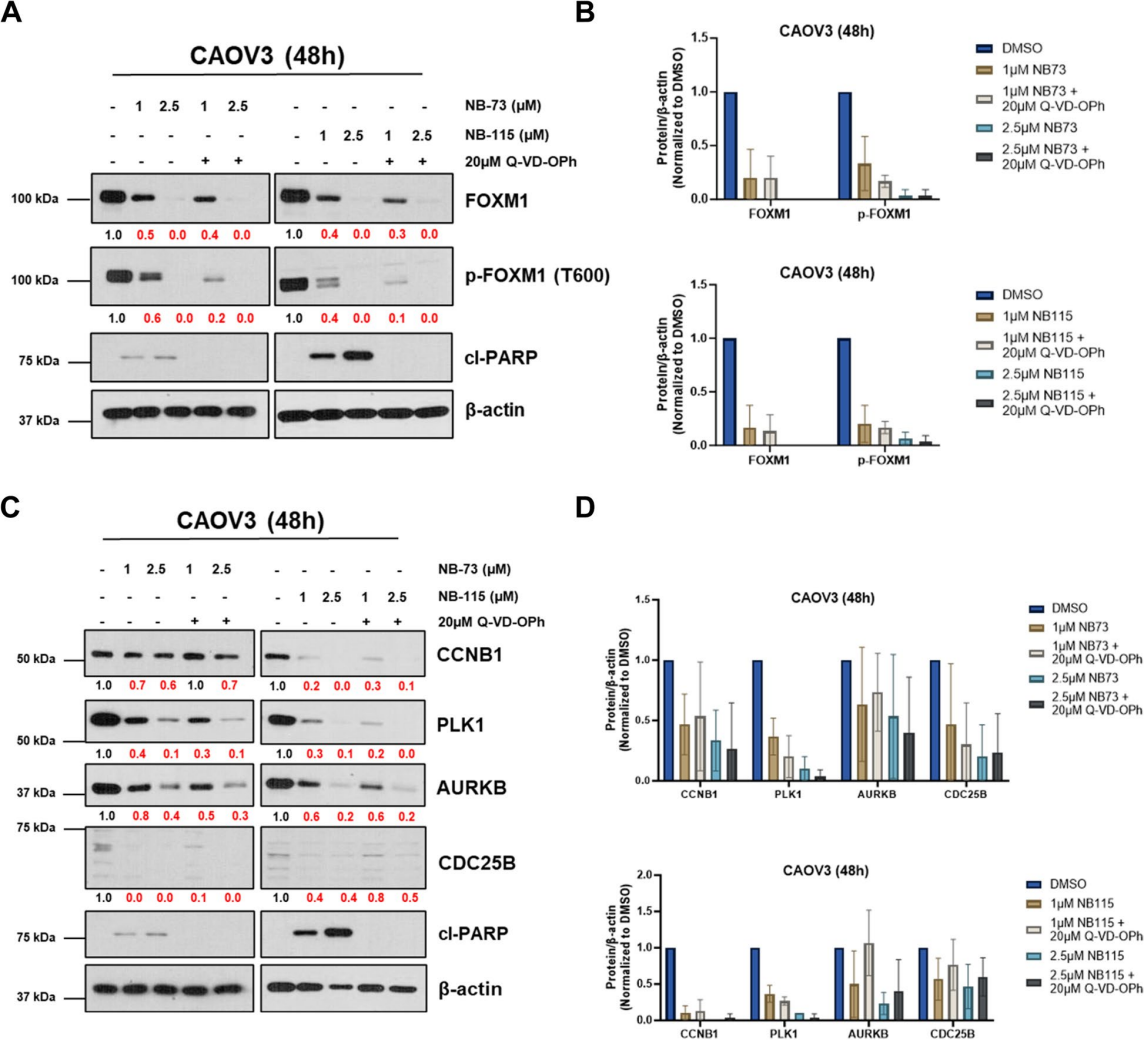
NB compound treatment mediated FOXM1 suppression is independent of apoptosis. **A** Western blot analysis of FOXM1, FOXM1-P, cl-PARP, and β-actin expression in CAOV3 cells treated with the indicated compounds for 48 h. Numerical values below the images indicate protein expression normalized by β-actin and red numbers indicate reduced expression relative to DMSO control. **B** Quantified western blot data from panel **A**; data from three biological replicates are plotted. Error bars indicate mean ± SD. **C** Western blot analysis of CCNB1, PLK1, AURKB, and CDC25B expression in CAOV3 cells treated with the indicated compounds for 48 h. **D** Quantified western blot data from panel **C**; data from three biological replicates are plotted. Error bars indicate mean ± SD

As NB compound treatment promotes apoptosis and apoptosis can lead to the degradation of cell signaling proteins [55, 56], we investigated whether FOXM1 suppression is a non-specific consequence of apoptosis in NB compound treated cells. For this, we measured FOXM1, FOXM1-P, and FOXM1 target expression after co-treatment of CAOV3 cells with NB compounds and the pan-caspase inhibitor Q-VD-OpH. Notably, FOXM1 and FOXM1-P remained suppressed after co-treatment (Fig. 10A and B). Furthermore, canonical FOXM1 targets exhibited the same behavior (Fig. 10C and D), and similar data were obtained for OVCAR4 cells (data not shown). Thus, the data support that FOXM1 pathway suppression is a primary outcome of NB compound treatment and is not an indirect consequence of apoptosis.

### NB compounds inhibit two-dimensional and three-dimensional HGSOC cell colony formation

**Fig.11:**
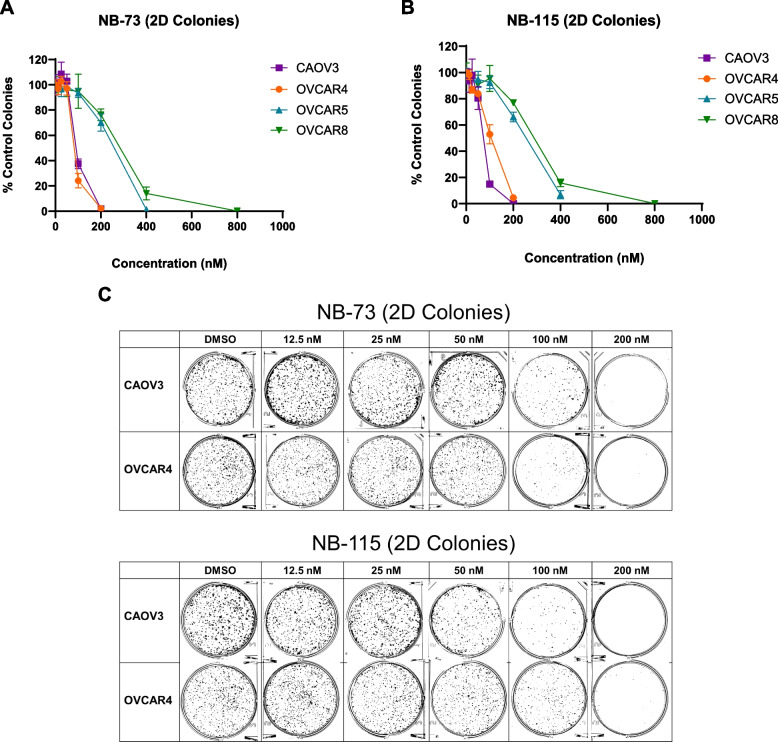
NB compound treatment inhibits 2D HGSOC colony formation. 2D colony formation of CAOV3, OVCAR4, OVCAR5, and OVCAR8 cells treated with (**A**) NB-73 or (**B**) NB-115 at the indicated concentrations. Values represent mean ± SD. **C** Representative images of 2D colony formation in CAOV3 and OVCAR4 cells treated with NB-73 or NB-115 at the indicated concentrations. Cell lines and drug concentrations are indicated

Two-dimensional (2D) anchorage-dependent colony formation is considered an in vitro assessment of self-renewing cells [57]. Based on data linking FOXM1 to tumor stem cell phenotypes [58], we examined the impact of NB compounds on this phenotype in four HGSOC cell lines: CAOV3, OVCAR4, OVCAR5, and OVCAR8. Notably, NB compounds restricted HSGOC 2D colony formation at sub-micromolar concentrations (Fig. 11).

**Fig.12:**
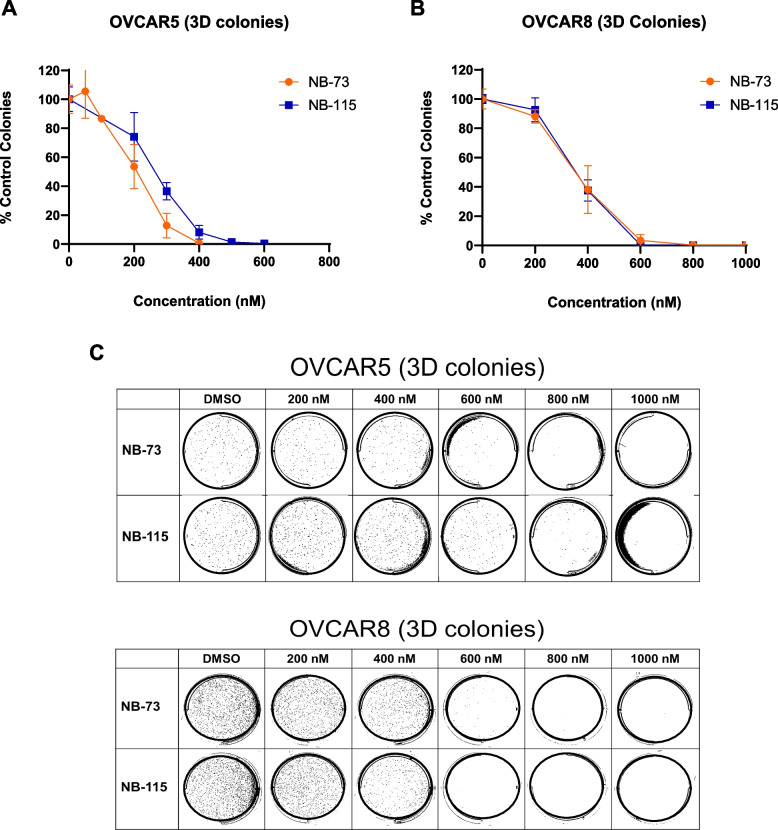
NB compound treatment inhibits 3D HGSOC colony formation. 3D colony formation of **A** OVCAR5 and **B** OVCAR8 cells treated with NB-73 or NB-115. Values represent mean ± SD. **C** Representative images of 3D colony formation in OVCAR5 and OVCAR8 cells treated with NB compounds at the indicated concentrations

Three-dimensional (3D) colony formation, an in vitro measure of anchorage-independent cell growth, is a classical measure of cellular transformation [59]. To test the effect of NB compounds on this phenotype, we utilized OVCAR5 and OVCAR8 HGSOC cells, which exhibited strong 3D growth compared to other HGSOC cell lines examined (data not shown). Similar to the outcome of the 2D colony formation assays, treatment of HGSOC cells with NB compounds inhibited 3D colony formation at sub-micromolar concentrations (Fig. 12).

### NB compounds synergize with carboplatin to inhibit HGSOC cell viability

To investigate the potential use of the NB compounds in combination with important HGSOC chemotherapies, we investigated combination treatments of NB-73 or NB-115 with carboplatin or the PARP inhibitor olaparib. Combinations with these agents are supported by the function of FOXM1 in stimulating the expression of DNA repair genes [7], as well as studies using genetic depletions of FOXM1 [60]. Notably, except for the carboplatin + NB-115 combination CAOV3 cells, carboplatin synergized with both NB compounds in both cell lines (Table 5). In contrast, the combination of NB compounds with olaparib was not synergistic (Table S1). To further examine the carboplatin combination, we used two different treatment regimens, the first in which NB compounds and carboplatin were administered to cells simultaneously, the second in which the NB compound was used first, and carboplatin was administered 24 h later. In both cases, we measured cell viability by CyQuant assay 72 h after the first treatment. Although the data were similar in both cases, the sequential regimen resulted in slightly greater synergy (Table 5).

**Table 5:** Combination Index testing of Carboplatin + NB compound treatment inHGSOC cell lines^a^

Combination	Cell Line	ED_75_	ED_90_	ED_95_	CI Average^b^
Carboplatin + NB-73 (Simultaneous Treatment)	CAOV3	1.09	**0.63**^c^	**0.44**^c^	**0.72**^c^
	OVCAR4	1.23	**0.75**^c^	**0.54**^c^	**0.84**^c^
Carboplatin + NB-115 (Simultaneous Treatment)	CAOV3	2.25	1.57	1.23	1.68
	OVCAR4	**0.94**^c^	**0.59**^c^	**0.43**^c^	**0.65**^c^
Carboplatin + NB-73 (Sequential Treatment)	CAOV3	1.10	**0.56**^c^	**0.36**^c^	**0.68**^c^
	OVCAR4	1.17	**0.60**^c^	**0.38**^c^	**0.72**^c^
Carboplatin + NB-115 (Sequential Treatment)	CAOV3	1.67	**0.95**^c^	**0.64**^c^	1.31
	OVCAR4	1.18	**0.50**^c^	**0.28**^c^	**0.65**^c^

^a^CyQuant assay measurement of cell viability loss

^b^The combination index (CI) of the effective doses (ED) for 75%, 90% and 95% of the population was averaged to create CI_average_, and synergy is defined as CI < 1, additivity as CI = 1, and antagonism as CI > 1

^c^Synergistic interactions indicated in bold font

## Discussion

FOXM1 is frequently overexpressed in HGSOC, which results in the activation of the FOXM1 pathway in almost 90% of primary HGSOC tumors [8, 9]. Additionally, we have recently shown that FOXM1 is expressed in chemoresistant recurrent HGSOC [60]. Importantly, FOXM1 promotes critical oncogenic phenotypes in HGSOC and other malignancies [7, 61]. These data support the evaluation of the therapeutic potential of FOXM1 inhibitors in HGSOC. We tested the in vitro activity of several FOXM1 inhibitors in terms of their potency, efficacy, and selectivity towards HGSOC cells, as compared to FTE cells [31, 32]. These investigations led to prioritization of NB-73 and NB-115 as the FOXM1 inhibitors that showed the most optimal combination of potency, efficacy, and selectivity. Subsequently, we carried out a series of investigations of the in vitro activity of these agents in HGSOC cell lines.

NB compounds suppressed FOXM1 and its targets in HGSOC cells and decreased HGSOC cell viability by promoting apoptosis. NB compounds have been shown to harbor similar activities in breast cancer cells (IC50 values ~ 1 μM) [29] and, consistent with earlier observations, the 1,1-diarylethylene diamine methiodide salts NB-73 and NB-115 were more potent FOXM1 inhibitors and suppressors of HGSOC cell viability than the 1,1-diarylethylene monoamine methiodide NB-55 [29]. Additionally, NB compounds suppress 2D and 3D HGSOC colony formation at sub-micromolar concentrations. As colonies form from a single cell, the data suggest the NB compounds might exert anti-cancer effects on tumor-initiating cells growing at metastatic sites, in addition to bulk cancer cells in the primary tumor.

In addition to FOXM1, NB compounds led to suppression of FOXA1 and FOXK2, but not FOXO3a. Although the decrease in expression of FOXA1 and FOXK2 were less dramatic than FOXM1, it is likely that these effects are biologically significant, and in part account for the anti-cancer activity of NB compounds. FOXA1 has oncogenic activity in ovarian cancer, and FOXK2 was recently identified as a promoter of HGSOC stem cell function [48, 62].

NB compounds induced apoptosis in HGSOC cell lines. We thus addressed whether NB compound mediated FOXM1 suppression is a consequence of apoptosis. After abrogation of apoptosis with a pan-caspase inhibitor, FOXM1 and FOXM1 target proteins remained suppressed. This result implicates FOXM1 suppression as a primary drug effect and not the result of NB compound mediated cytotoxicity. This is consistent with prior observations showing that NB compounds directly bind to FOXM1 in cells, leading to its proteolytic degradation [29].

Interestingly, drug washout experiments indicated that the effect of NB compounds on FOXM1 lasts for up to three days following drug removal. These results point towards a relatively stable PD effect that may be considered a favorable characteristic of these agents. These in vitro data are consistent with the extended half-life of these agents seen in vivo in mice [29].

Two of the standard chemotherapies used in clinical management of HGSOC are carboplatin and olaparib [1]. Based on the role of FOXM1 in activating DNA repair genes [7, 61], we investigated the potential for combination treatments of NB compounds with these two agents. Interestingly, there was a concentration-dependent synergistic interaction with carboplatin, but not olaparib. These data suggest that NB compounds might have utility in combination treatment with DNA damaging agents in HGSOC, but not with PARP inhibitors such as olaparib, which do not directly damage DNA. However, further research is needed to clarify the mechanistic basis for this distinction between carboplatin and PARP inhibitors.

This study has two key limitations. First, we did not investigate the efficacy of NB compounds in vivo using HGSOC models, which is an important area for future research. However, we note the in vivo activity of NB compounds has been reported in other tumor types, including breast, melanoma, and multiple myeloma [63–65]. Second, we determined NB compound targets and cellular effects mainly based on prior knowledge, as opposed to an unbiased assessment. Previously, RNA-seq (an unbiased approach) was used to explore the cellular effects of NB compounds in breast cancer cells, which identified several interesting biochemical pathways that were affected [66]. Analogous studies should now be conducted in the context of HGSOC cells to expand our knowledge of the molecular action of NB compounds in HGSOC.

## Conclusions

Among a panel of FOXM1 inhibitors tested, NB-73 and NB-115 displayed the most optimal combination of potency, efficacy, and selectivity for HGSOC cells. NB compounds effectively suppressed FOXM1, transcriptionally active FOXM1, and canonical FOXM1 target genes in HGSOC cells. NB compounds promote FOXM1 protein degradation in HGSOC cells, and the primary cellular outcome of NB compound treatment was apoptosis. Suppression of FOXM1 continued in the presence of a pan-caspase inhibitor, suggesting that FOXM1 suppression is a direct drug effect. The suppressive effects of NB compounds on FOXM1 lasted for up to 72 h following one drug exposure. NB compounds suppressed HGSOC colony formation in 2D and 3D culture and synergized with carboplatin in HGSOC cells. In summary, NB-73 and NB-115 are potent and efficacious FOXM1 inhibitors in HGSOC cells.

### Supplementary Information

**Supplementary Material 1. **

**Supplementary Material 2. **

## Data Availability

The datasets used and/or analyzed during the current study are available from the corresponding author on reasonable request.

## Abbreviations

2D: Two dimensional
3D: Three dimensional
AURKB: Aurora Kinase B
CCNB1: Cyclin B1
CDC25B: Cell Division Cycle 25B
cl-PARP: Cleaved PARP
DBD: DNA binding domain
FBS: Fetal bovine serum
FDI-6: Forkead domain inhibitor 6
FOXA1: Forkhead box A1
FOXK2: Forkhead box K2
FOXO3a: Forkead box O3a
FOXM1: Forkhead box M1
FTE: Fallopian tube epithelium
HGSOC: High grade serous ovarian cancer
IC50: 50% Inhibitory concentration
NB compounds: NB 73 and NB115
PARP: Poly ADP ribose polymerase
PD: Pharmacodynamic
Pen-strep: Penicillin–streptomycin
PLK1: Polo Like Kinase 1
PROTAC: Proteolysis-targeting chimera
RCM-1: Robert Costa memorial compound 1
RIPA: Radioimmune Assay
RT-qPCR: Reverse transcriptase quantitative PCR
SEM: Standard error of the mean
SD: Standard deviation
TNBC: Triple negative breast cancer

## References

[1] Bowtell DD, Bohm S, Ahmed AA, Aspuria PJ, Bast RC, Beral V, Berek JS, Birrer MJ, Blagden S, Bookman MA (2015). Rethinking ovarian cancer II: reducing mortality from high-grade serous ovarian cancer. Nat Rev Cancer.

[2] Laoukili J, Stahl M, Medema RH (2007). FoxM1: at the crossroads of ageing and cancer. Biochem Biophys Acta.

[3] Raychaudhuri P, Park HJ (2011). FoxM1: a master regulator of tumor metastasis. Can Res.

[4] Alvarez-Fernández M, Medema RH (2013). Novel functions of FoxM1: from molecular mechanisms to cancer therapy. Front Oncol.

[5] Halasi M, Gartel AL (2013). FOX(M1) news–it is cancer. Mol Cancer Ther.

[6] Lam EW, Brosens JJ, Gomes AR, Koo CY (2013). Forkhead box proteins: tuning forks for transcriptional harmony. Nat Rev Cancer.

[7] Liu C, Barger CJ, Karpf AR (2021). FOXM1: A Multifunctional Oncoprotein and Emerging Therapeutic Target in Ovarian Cancer. Cancers.

[8] Bell D, Berchuck A, Birrer M, Chien J, Cramer DW, Dao F, Dhir R, DiSaia P, Gabra H, Glenn P (2011). Integrated genomic analyses of ovarian carcinoma. Nature.

[9] Barger CJ, Zhang W, Hillman J, Stablewski AB, Higgins MJ, Vanderhyden BC, Odunsi K, Karpf AR (2015). Genetic determinants of FOXM1 overexpression in epithelial ovarian cancer and functional contribution to cell cycle progression. Oncotarget.

[10] Zhang Q, Zhang R, Liu M, Wu H, Yang B (2023). An integrated model of CDCA5 and FOXM1 expression combined with a residual disease that predicts prognosis in ovarian cancer patients. Cell Mol Biol (Noisy-le-grand).

[11] Sousa A, Dugourd A, Memon D, Petursson B, Petsalaki E, Saez-Rodriguez J, Beltrao P (2023). Pan-Cancer landscape of protein activities identifies drivers of signalling dysregulation and patient survival. Mol Syst Biol.

[12] Barger CJ, Branick C, Chee L, Karpf AR (2019). Pan-cancer analyses reveal genomic features of FOXM1 overexpression in cancer. Cancers (Basel).

[13] Raghuwanshi S, Gartel AL (2023). Small-molecule inhibitors targeting FOXM1: Current challenges and future perspectives in cancer treatments. Biochim Biophys Acta Rev Cancer.

[14] Kwok JM-M, Myatt SS, Marson CM, Coombes RC, Constantinidou D, Lam EW-F (2008). Thiostrepton selectively targets breast cancer cells through inhibition of forkhead box M1 expression. Mol Cancer Ther.

[15] Halasi M, Gartel AL (2009). A novel mode of FoxM1 regulation: Positive auto-regulatory loop. Cell Cycle.

[16] Hegde NS, Sanders DA, Rodriguez R, Balasubramanian S (2011). The transcription factor FOXM1 is a cellular target of the natural product thiostrepton. Nat Chem.

[17] Gartel A. Thiazole Antibiotics Siomycin a and Thiostrepton Inhibit the Transcriptional Activity of FOXM1. Front Oncol 2013, 3(150). 10.3389/fonc.2013.00150 PMC3674410 23761863

[18] Halasi M, Váraljai R, Benevolenskaya E, Gartel AL (2016). A novel function of molecular chaperone HSP70: suppression of oncogenic FOXM1 after proteotoxic stress *. J Biol Chem.

[19] Gormally MV, Dexheimer TS, Marsico G, Sanders DA, Lowe C, Matak-Vinković D, Michael S, Jadhav A, Rai G, Maloney DJ (2014). Suppression of the FOXM1 transcriptional programme via novel small molecule inhibition. Nat Commun.

[20] Chen X, Müller GA, Quaas M, Fischer M, Han N, Stutchbury B, Sharrocks AD, Engeland K (2013). The Forkhead Transcription Factor FOXM1 Controls Cell Cycle-Dependent Gene Expression through an Atypical Chromatin Binding Mechanism. Mol Cell Biol.

[21] Iness AN, Litovchick L (2018). MuvB: A Key to Cell Cycle Control in Ovarian Cancer. Front Oncol.

[22] Sanders DA, Gormally MV, Marsico G, Beraldi D, Tannahill D, Balasubramanian S (2015). FOXM1 binds directly to non-consensus sequences in the human genome. Genome Biol.

[23] Kang K, Choi Y, Kim HH, Yoo KH, Yu S (2020). Predicting FOXM1-Mediated Gene Regulation through the Analysis of Genome-Wide FOXM1 Binding Sites in MCF-7, K562, SK-N-SH, GM12878 and ECC-1 Cell Lines. Int J Mol Sci.

[24] Tang Q, Liu C, Zhang S, He L, Liu Y, Wang J, Zhao X, Li X (2023). FOXM1 increases hTERT protein stability and indicates poor prognosis in gastric cancer. Neoplasia.

[25] Zhang N, Wei P, Gong A, Chiu WT, Lee HT, Colman H, Huang H, Xue J, Liu M, Wang Y (2011). FoxM1 promotes beta-catenin nuclear localization and controls Wnt target-gene expression and glioma tumorigenesis. Cancer Cell.

[26] Ketola K, Munuganti RSN, Davies A, Nip KM, Bishop JL, Zoubeidi A (2017). Targeting Prostate Cancer Subtype 1 by Forkhead Box M1 Pathway Inhibition. Clin Cancer Res.

[27] Chen Y, Ruben EA, Rajadas J, Teng NNH (2015). In silico investigation of FOXM1 binding and novel inhibitors in epithelial ovarian cancer. Bioorg Med Chem.

[28] Sun L, Ren X, Wang I-C, Pradhan A, Zhang Y, Flood HM, Han B, Whitsett JA, Kalin TV, Kalinichenko VV (2017). The FOXM1 inhibitor RCM-1 suppresses goblet cell metaplasia and prevents IL-13 and STAT6 signaling in allergen-exposed mice. Sci Signaling.

[29] Ziegler Y, Laws MJ, Sanabria Guillen V, Kim SH, Dey P, Smith BP, Gong P, Bindman N, Zhao Y, Carlson K (2019). Suppression of FOXM1 activities and breast cancer growth in vitro and in vivo by a new class of compounds. nbj Breast Cancer.

[30] Koboldt DC, Fulton RS, McLellan MD, Schmidt H, Kalicki-Veizer J, McMichael JF, Fulton LL, Dooling DJ, Ding L, Mardis ER (2012). Comprehensive molecular portraits of human breast tumours. Nature.

[31] Perets R, Drapkin R (2016). It's totally tubular riding the new wave of ovarian cancer research. Cancer Res.

[32] Klinkebiel D, Zhang W, Akers SN, Odunsi K, Karpf AR (2016). DNA methylome analyses implicate Fallopian Tube Epithelia as the origin for high-grade serous ovarian cancer. Mol Cancer Res.

[33] Karst AM, Jones PM, Vena N, Ligon AH, Liu JF, Hirsch MS, Etemadmoghadam D, Bowtell DDL, Drapkin R (2014). Cyclin E1 Deregulation Occurs Early in Secretory Cell Transformation to Promote Formation of Fallopian Tube-Derived High-Grade Serous Ovarian Cancers. Can Res.

[34] Barger CJ, Zhang W, Sharma A, Chee L, James SR, Kufel CN, Miller A, Meza J, Drapkin R, Odunsi K (2018). Expression of the POTE gene family in human ovarian cancer. Sci Rep.

[35] Schindelin J, Arganda-Carreras I, Frise E, Kaynig V, Longair M, Pietzsch T, Preibisch S, Rueden C, Saalfeld S, Schmid B (2012). Fiji: an open-source platform for biological-image analysis. Nat Methods.

[36] Brzozowska B, Gałecki M, Tartas A, Ginter J, Kaźmierczak U, Lundholm L (2019). Freeware tool for analysing numbers and sizes of cell colonies. Radiat Environ Biophys.

[37] Cokol-Cakmak M, Bakan F, Cetiner S, Cokol M (2018). Diagonal Method to Measure Synergy Among Any Number of Drugs. JoVE.

[38] Domcke S, Sinha R, Levine DA, Sander C, Schultz N (2013). Evaluating cell lines as tumour models by comparison of genomic profiles. Nat Commun.

[39] Mitra AK, Davis DA, Tomar S, Roy L, Gurler H, Xie J, Lantvit DD, Cardenas H, Fang F, Liu Y (2015). In vivo tumor growth of high-grade serous ovarian cancer cell lines. Gynecol Oncol.

[40] Laoukili J, Alvarez M, Meijer LAT, Stahl M, Mohammed S, Kleij L, Heck AJR, Medema RH (2008). Activation of FoxM1 during G2 Requires Cyclin A/Cdk-Dependent Relief of Autorepression by the FoxM1 N-Terminal Domain. Mol Cell Biol.

[41] Anders L, Ke N, Hydbring P, Choi YJ, Widlund HR, Chick JM, Zhai H, Vidal M, Gygi SP, Braun P (2011). A systematic screen for CDK4/6 substrates links FOXM1 phosphorylation to senescence suppression in cancer cells. Cancer Cell.

[42] Wang X, Quail E, Hung N-J, Tan Y, Ye H, Costa RH (2001). Increased levels of forkhead box M1B transcription factor in transgenic mouse hepatocytes prevent age-related proliferation defects in regenerating liver. Proc Natl Acad Sci.

[43] Leung TWC, Lin SSW, Tsang ACC, Tong CSW, Ching JCY, Leung WY, Gimlich R, Wong GG, Yao K-M (2001). Over-expression of FoxM1 stimulates cyclin B1 expression. FEBS Lett.

[44] Laoukili J, Kooistra MRH, Brás A, Kauw J, Kerkhoven RM, Morrison A, Clevers H, Medema RH (2005). FoxM1 is required for execution of the mitotic programme and chromosome stability. Nat Cell Biol.

[45] Bi X, Zheng D, Cai J, Xu D, Chen L, Xu Z, Cao M, Li P, Shen Y, Wang H (2023). Pan-cancer analyses reveal multi-omic signatures and clinical implementations of the forkhead-box gene family. Cancer Med.

[46] Wang L-L, Xiu Y-L, Chen X, Sun K-X, Chen S, Wu D-D, Liu B-L, Zhao Y (2017). The transcription factor FOXA1 induces epithelial ovarian cancer tumorigenesis and progression. Tumor Biology.

[47] Wang K, Guan C, Fang C, Jin X, Yu J, Zhang Y, Zheng L (2018). Clinical significance and prognostic value of Forkhead box A1 expression in human epithelial ovarian cancer. Oncol Lett.

[48] Zhang Y, Wang Y, Zhao G, Tanner EJ, Adli M, Matei D (2022). FOXK2 promotes ovarian cancer stemness by regulating the unfolded protein response pathway. J Clin Invest.

[49] Liu Y, Ao X, Ding W, Ponnusamy M, Wu W, Hao X, Yu W, Wang Y, Li P, Wang J (2018). Critical role of FOXO3a in carcinogenesis. Mol Cancer.

[50] Tonge PJ (2018). Drug-Target Kinetics in Drug Discovery. ACS Chem Neurosci.

[51] Peper A (2009). Aspects of the relationship between drug dose and drug effect. Dose Response.

[52] Ward TH, Cummings J, Dean E, Greystoke A, Hou JM, Backen A, Ranson M, Dive C (2008). Biomarkers of apoptosis. Br J Cancer.

[53] Chaitanya GV, Steven AJ, Babu PP (2010). PARP-1 cleavage fragments: signatures of cell-death proteases in neurodegeneration. Cell Commun Signal.

[54] Caserta TM, Smith AN, Gultice AD, Reedy MA, Brown TL (2003). Q-VD-OPh, a broad spectrum caspase inhibitor with potent antiapoptotic properties. Apoptosis.

[55] Widmann C, Gibson S, Johnson GL (1998). Caspase-dependent cleavage of signaling proteins during apoptosis A turn-off mechanism for anti-apoptotic signals. J Biol Chem.

[56] Sukharev SA, Pleshakova OV, Sadovnikov VB (1997). Role of proteases in activation of apoptosis. Cell Death Differ.

[57] Brix N, Samaga D, Belka C, Zitzelsberger H, Lauber K (2021). Analysis of clonogenic growth in vitro. Nat Protoc.

[58] Sher G, Masoodi T, Patil K, Akhtar S, Kuttikrishnan S, Ahmad A, Uddin S (2022). Dysregulated FOXM1 signaling in the regulation of cancer stem cells. Semin Cancer Biol.

[59] Borowicz S, Van Scoyk M, Avasarala S, Karuppusamy Rathinam MK, Tauler J, Bikkavilli RK, Winn RA (2014). The soft agar colony formation assay. J Vis Exp.

[60] Barger CJ, Chee L, Albahrani M, Munoz-Trujillo C, Boghean L, Branick C, Odunsi K, Drapkin R, Zou L, Karpf AR (2021). Co-regulation and function of FOXM1/RHNO1 bidirectional genes in cancer. eLife.

[61] Kalathil D, John S, Nair AS (2020). FOXM1 and Cancer: Faulty Cellular Signaling Derails Homeostasis. Front Oncol.

[62] Wang LL, Xiu YL, Chen X, Sun KX, Chen S, Wu DD, Liu BL, Zhao Y (2017). The transcription factor FOXA1 induces epithelial ovarian cancer tumorigenesis and progression. Tumour Biol.

[63] Doepner M, Lee I, Natale CA, Brathwaite R, Venkat S, Kim SH, Wei Y, Vakoc CR, Capell BC, Katzenellenbogen JA (2022). Endogenous DOPA inhibits melanoma through suppression of CHRM1 signaling. Sci Adv.

[64] Cheng Y, Sun F, Thornton K, Jing X, Dong J, Yun G, Pisano M, Zhan F, Kim SH, Katzenellenbogen JA (2022). FOXM1 regulates glycolysis and energy production in multiple myeloma. Oncogene.

[65] Nandi I, Smith HW, Sanguin-Gendreau V, Ji L, Pacis A, Papavasiliou V, Zuo D, Nam S, Attalla SS, Kim SH (2023). Coordinated activation of c-Src and FOXM1 drives tumor cell proliferation and breast cancer progression. J Clin Invest.

[66] Ziegler Y, Guillen VS, Kim SH, Katzenellenbogen JA, Katzenellenbogen BS (2021). Transcription Regulation and Genome Rewiring Governing Sensitivity and Resistance to FOXM1 Inhibition in Breast Cancer. Cancers (Basel).

