# Supplementary material for: NB compounds are potent and efficacious FOXM1 inhibitors in high-grade serous ovarian cancer cells
Source: J Ovarian Res. 2024 May 4;17:94. doi: 10.1186/s13048-024-01421-4 (PMC11069232; doi:10.1186/s13048-024-01421-4)
Supplement: Supplementary file 1 — Supplementary Material 1. [file 13048_2024_1421_MOESM1_ESM.pptx]

## Slide 1
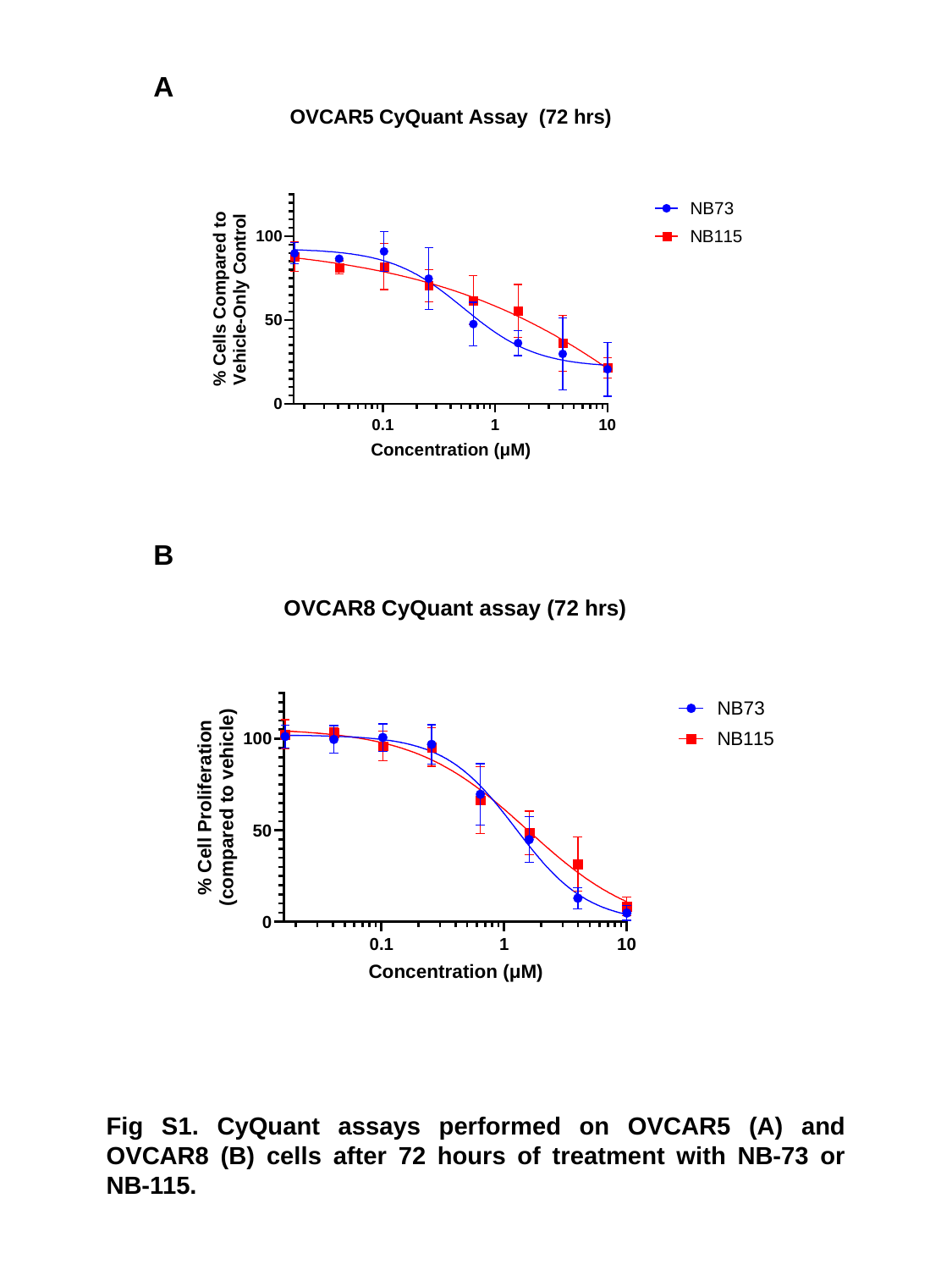

A
B
Fig S1. CyQuant assays performed on OVCAR5 (A) and OVCAR8 (B) cells after 72 hours of treatment with NB-73 or NB-115.

## Slide 2
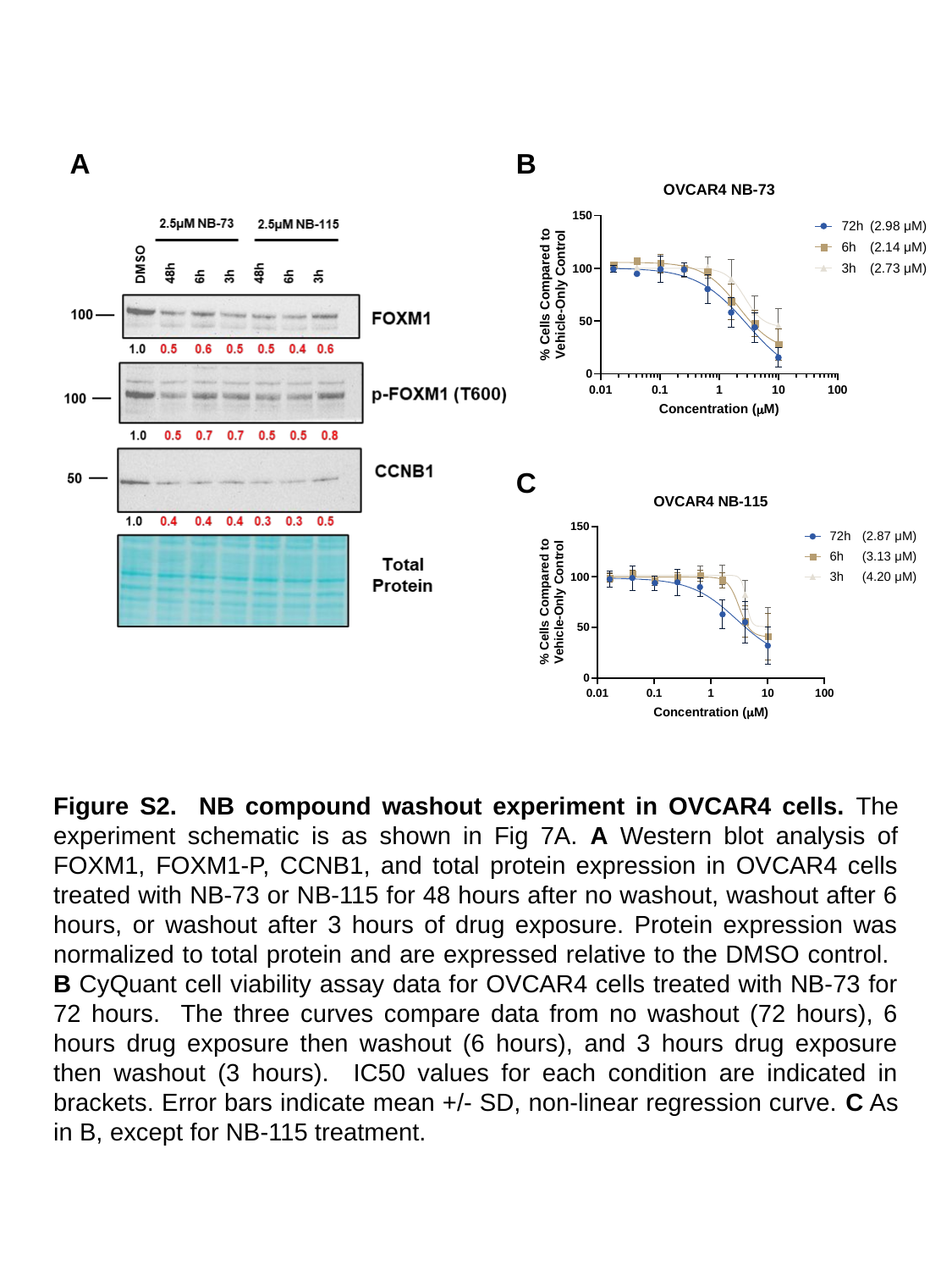

A
B
C
Figure S2. NB compound washout experiment in OVCAR4 cells. The experiment schematic is as shown in Fig 7A. A Western blot analysis of FOXM1, FOXM1-P, CCNB1, and total protein expression in OVCAR4 cells treated with NB-73 or NB-115 for 48 hours after no washout, washout after 6 hours, or washout after 3 hours of drug exposure. Protein expression was normalized to total protein and are expressed relative to the DMSO control. B CyQuant cell viability assay data for OVCAR4 cells treated with NB-73 for 72 hours. The three curves compare data from no washout (72 hours), 6 hours drug exposure then washout (6 hours), and 3 hours drug exposure then washout (3 hours). IC50 values for each condition are indicated in brackets. Error bars indicate mean +/- SD, non-linear regression curve. C As in B, except for NB-115 treatment.

## Slide 3
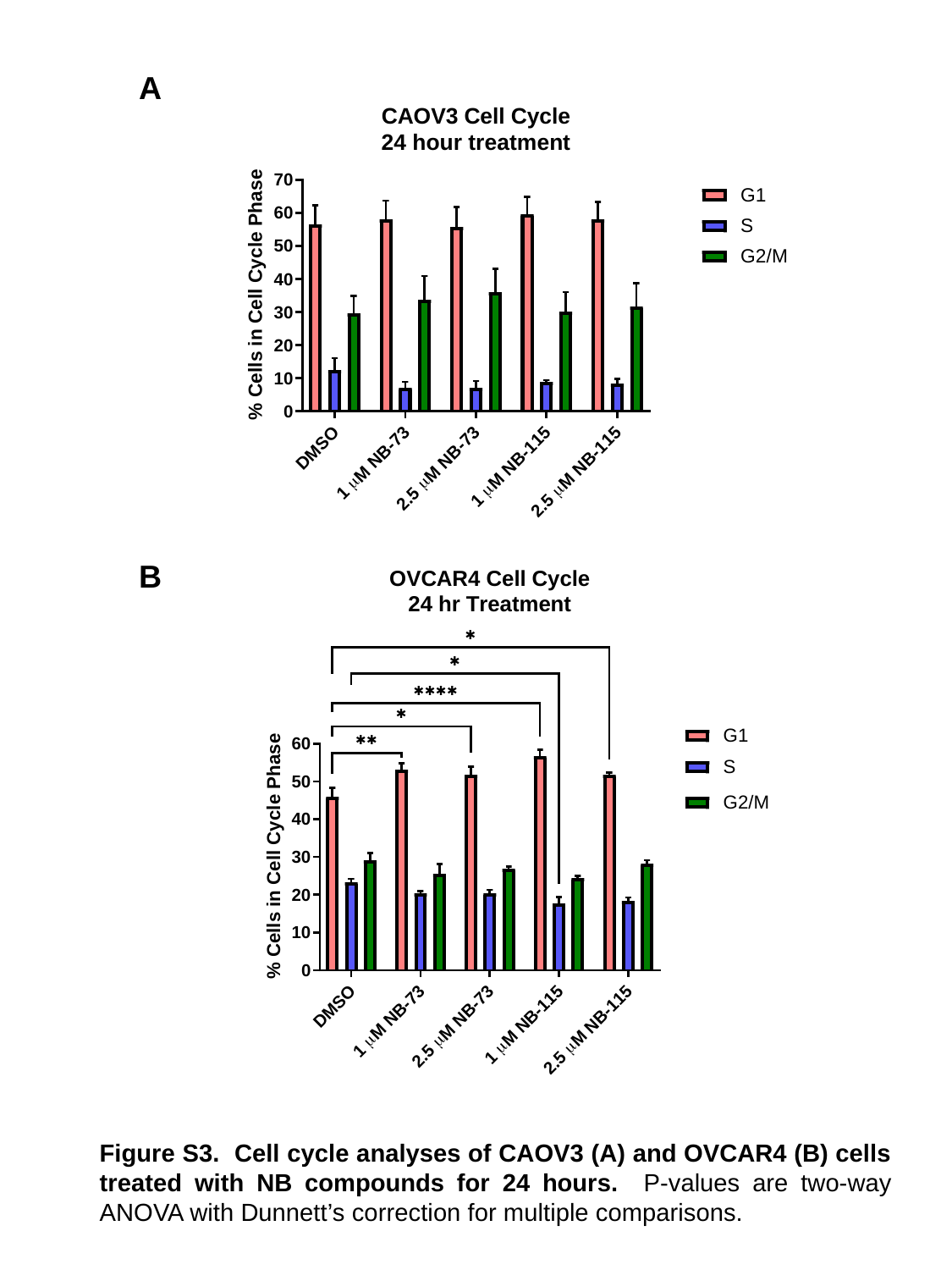

A
B
Figure S3. Cell cycle analyses of CAOV3 (A) and OVCAR4 (B) cells treated with NB compounds for 24 hours. P-values are two-way ANOVA with Dunnett’s correction for multiple comparisons.
