# Supplementary material for: NB compounds are potent and efficacious FOXM1 inhibitors in high-grade serous ovarian cancer cells
Source: J Ovarian Res. 2024 May 4;17:94. doi: 10.1186/s13048-024-01421-4 (PMC11069232; doi:10.1186/s13048-024-01421-4)
Supplement: Supplementary file 2 — Supplementary Material 2. [file 13048_2024_1421_MOESM2_ESM.docx]

**Table S1. Combination Index testing of olaparib + NB compounds in HGSOC cell lines.**

| **Drug Combination^1^** | **HGSOC Cell Line** | **ED_75_** | **ED_90_** | **ED_95_** | **CI Average** |
| --- | --- | --- | --- | --- | --- |
| Olaparib + NB-73 | CAOV3 | 5.34 | 3.60 | 2.75 | 3.90 |
|  | OVCAR4 | 1.26 | 1.41 | 1.52 | 1.40 |
| Olaparib + NB-115 | CAOV3 | 2.15 | 2.24 | 2.31 | 2.23 |
|  | OVCAR4 | 1.36 | 0.92 | 0.71 | 1.00 |

**^1^ Cells were treated with both drugs simultaneously.**
